# Exploring the Impact of Osteopathic Manipulative Treatment on Chronic Migraine Relief: A Case Study

**DOI:** 10.7759/cureus.78485

**Published:** 2025-02-04

**Authors:** Yassine Lahlou, Mariam Aamir, Victoria Shadiack

**Affiliations:** 1 Osteopathic Manipulative Medicine, Edward Via College of Osteopathic Medicine, Spartanburg, USA; 2 Family Medicine/Osteopathic Neuromusculoskeletal Medicine (ONMM), Edward Via College of Osteopathic Medicine, Spartanburg, USA

**Keywords:** activities of daily life, failed medication therapy, headache relief, manual modality, migraine-type headache, non-pharmacological treatment, osteopathic manipulative medicine (omm), osteopathic manipulative treatment (omt), preventive intervention, refractory headache

## Abstract

For years, migraines have been the subject of extensive research, as their symptoms significantly burden individuals’ quality of life. Existing treatments primarily rely on pharmaceutical interventions targeting various symptoms associated with migraines. However, the varying efficacy and side effects of these medications have led to the consideration of alternative treatment modalities to improve patient outcomes and minimize adverse effects. This case study investigates the efficacy of osteopathic manipulative treatment (OMT) for the management of chronic migraines in a 27-year-old female patient. This patient presented with a history of debilitating migraines with auras that began in 2018. She had previously tried various medications for migraine prophylaxis with limited success. After years of poorly controlled migraines, she decided to enroll in a clinical trial that required an abrupt discontinuation of her previous treatment involving a medication washout period. During this time, her symptoms worsened and significantly impacted her ability to perform activities of daily living (ADL). Additionally, during this time, she received a single OMT treatment. Following this treatment, she reported immediate improvement, including a reduction in migraine frequency and elimination of nausea and visual auras. This case study highlights the benefit of incorporating OMT as an effective treatment for chronic migraines, providing substantial relief and symptom reduction for one month. The incorporation of OMT alongside pharmacological treatments may provide comprehensive care to patients suffering from chronic migraines, thus enhancing their quality of life and alleviating the global burden of this medical condition.

## Introduction

Chronic migraines are headache disorders that are primarily characterized as recurrent throbbing or pulsing headaches that can also consist of systemic symptoms, including nausea, movement sensitivity, photophobia, and sensory auras. Migraines pose a significant detriment to the US population, affecting roughly 12% of the population and disproportionately affecting women at a 3:1 ratio compared to men [1]. It also affects people in the most productive period of life, between the ages of 25 and 55 years [1]. In 2015, migraines were the leading cause of neurological disability in the USA, causing a greater than 50% reduction in work productivity in affected individuals [2]. According to the US Headache Consortium, a treatment plan should be focused on treating attacks to prevent recurrence, improve one’s functioning ability, reduce usage of rescue medications, be cost-effective, and have minimal side effects [2].

Pharmacological interventions for chronic migraines encompass a range of medications. Treatment is focused on a twofold approach: acute abortive management and prophylactic prevention [2]. Pain relievers such as nonsteroidal anti-inflammatory drugs (NSAIDs) and triptans are used to provide immediate pain relief; NSAIDs are the most commonly used drugs in the treatment of acute attacks [3]. Preventive medications such as beta-blockers, antidepressants, and anticonvulsants aim to reduce the intensity and frequency of migraine episodes [4]. Although these pharmaceutical interventions can be effective in some individuals, they can also cause various side effects, including gastrointestinal (GI) symptoms such as epigastric pain, nausea, GI bleeding, and medication overuse headaches [3]. Furthermore, many patients may present with intolerance or may not respond well.

As our understanding of chronic migraines expands, it becomes apparent that research for a more comprehensive treatment plan is necessary. An alternative avenue for treatment is osteopathic manipulative medicine (OMM). Osteopathic manipulative medicine’s specific treatment modality, osteopathic manipulative treatment (OMT), is a non-pharmacological treatment that complements traditional medical care. Osteopathic manipulative treatment is a hands-on intervention focusing primarily on manipulating the musculoskeletal system to restore anatomical and physiological balance within the body. This technique is cost-effective compared to pharmaceuticals and has limited side effects, primarily temporary mild soreness [1]. Osteopathic physicians are trained to manage chronic pain with OMT to treat the root issue rather than just symptoms. This treatment plan considers the human body's interconnected nature and the biopsychosocial model of medicine [5].

Various studies have shown that OMT effectively alleviates pain and reduces it for approximately four to six weeks [5]. Due to this finding, some individuals may find better relief by combining pharmacologic interventions and OMT. This may provide a more comprehensive treatment plan that offers improved symptom management and a higher quality of life. One systematic review identified promising evidence showcasing OMT efficacy in reducing pain intensity, pain frequency, and decreasing disability [6].

This article was previously presented as a poster at the 2023 Edward Via College of Osteopathic Medicine - Carolinas Research Day on February 9, 2023.

## Case presentation

History of present illness

**Figure 1:**
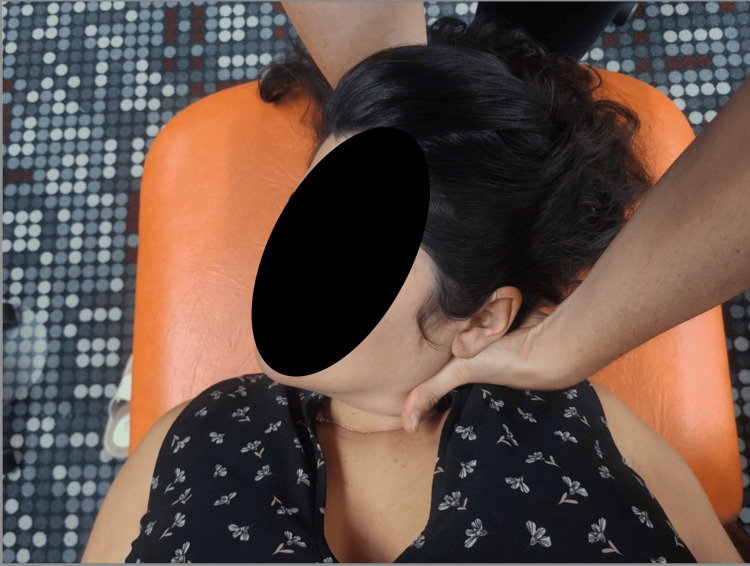
Cervical high-velocity low-amplitude (HVLA) with sidebending emphasis The patient is placed in the opposite position of the sidebending diagnosis, and a thrust is initiated through rotation in the same direction as the diagnosis. Photo Credit: Edward Via College of Osteopathic Medicine (VCOM), Bethany Powers, Doctor of Osteopathic Medicine (D.O.), and Victoria Shadiack D.O.

**Figure 2:**
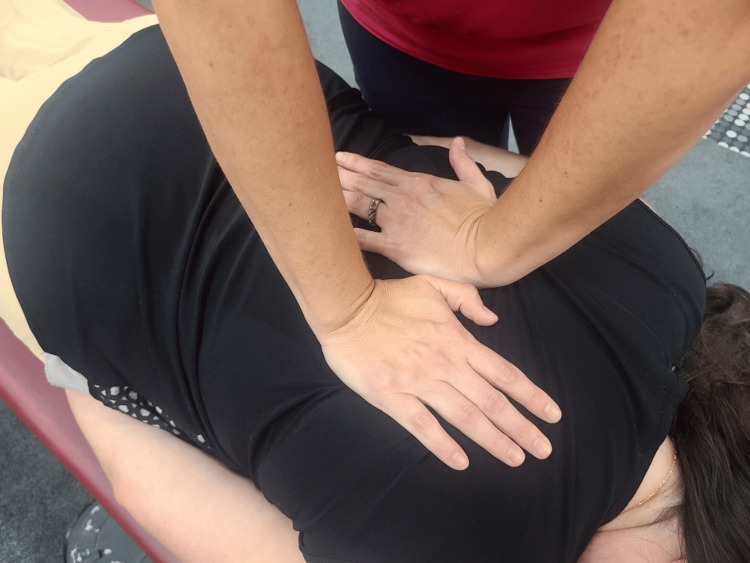
Texas twist The patient is placed in the prone position. The practitioner positions their hands in the direction of the restrictive barrier (opposite of osteopathic diagnosis), and a high-velocity low-amplitude (HVLA) corrective thrust is applied.

A 27-year-old female presented with complaints of worsening chronic migraines with visual and auditory auras that began in 2018. She described the migraines as debilitating to her normal activities of daily living (ADLs)-including walking, reading, watching TV, and driving. Her main recurrent symptoms included nausea and head tightness. She noted that her main trigger was stress. Her migraine frequency was three to five days/week, with a reported 0-10 pain scale ranging from 8/10 to 10/10. She mentioned using a non-pharmacologic treatment of a compression ice hat, which provided temporary moderate pain relief. Pharmacologic therapy included propranolol, duloxetine Hcl (Cymbalta, Eli Lilly and Company, Indianapolis, IN), and sumatriptan (Imitrex, GlaxoSmithKline, Durham, NC), all of which provided varying results. She reported a mixed response to pharmaceutical intervention. Initially, she experienced increased migraine symptoms and adverse side effects. However, this was followed by a reduction in migraine severity to 6/10 and a decrease in migraine frequency to two to four days per week for the last three years until January 2023. By January 2023, her migraines were no longer adequately managed by medications, and she subsequently enrolled in a clinical trial for a new migraine drug. This required a medication wash-out period, which caused migraine severity and frequency to return to her off-medication baseline. She presented for a single OMT intervention during the first week of the wash-out period; a focused review of symptoms was conducted (Table 1). Immediately before OMT, she described her migraine as “severe, pounding, twisting, and pulsating.” Her pain was located in her frontal bone and radiated to her ears bilaterally. The patient reported no significant family history or surgical history. Her past medical history was positive for complex regional pain syndrome. An osteopathic examination was completed, and she was treated accordingly (Table 2). The treatments were primarily focused on the head, cervical, and thoracic regions with techniques including front lift, counterstrain, cervical high-velocity low-amplitude (HVLA) (Figure 1), and Texas twist (Figure 2). 

**Table 1:** Symptomatic manifestations of the patient's migraine Note: The patient's symptoms are described through a focused review of symptoms)​​​​

The patient's migraine symptpms	
General	Fatigue
Head	Headache and dizziness
Ears	Tinnitus
Eyes	Diplopia, pulsatile vision, and blurry vision
Neuro	Numbness, tingling, and paresthesia
Gastrointestinal (GI)	Nausea, diarrhea, indigestion, and heartburn

**Table 2:** Osteopathic structural exam and techniques utilized OMT: osteopathic manipulative treatment; C2: cervical vertebra 2; FRSl: flexed, rotated, and sidebent left; C4: cervical vertebra 4; HVLA: high-velocity low amplitude; T1-3: thoracic vertebra 1,2, and 3; NRlSr: neutral, rotated left; sidebent right

Body region	Somatic dysfunction	OMT performed
Head	Falx cerebri restriction on midline sagittal suture	Frontal lift, orbital lift with nasion distraction
Cervical	C2 FRSl C4 FRSl; bilateral sternocleidomastoid tender point	HVLA (Figure 1); Counterstrain
Thoracic	T1-3 Type 1 grouped, NRlSr	HVLA: Texas twist (Figure 2)
Other body regions were not evaluated for this single OMT intervention		

Results

At the end of the OMT intervention, an acute reduction in the severity of symptoms was noted from 9.5/10 to 5/10. The patient was evaluated after the treatment at time intervals of one week, one month, and six months. She did not receive any additional OMT during the time intervals of one week and one month. At her one-week evaluation, she described being migraine-free for the first three days following treatment. Her migraine episode frequency decreased to one day per week, and her pain character changed from a descriptor of “severe pounding” to “aching.” Her one-day-per-week migraine had no visual or auditory auras compared to baseline. She also reported that she was able to return to her ADLs without her common nausea symptoms. At her one-month evaluation, she noted beginning her clinical trial with an undisclosed monotherapy due to a nondisclosure agreement (NDA). At that time her migraine frequency increased to two days per week, and her pain character was unchanged from “aching”. She noted no return of visual or auditory auras, but her symptoms were significant for brain fog and nausea. At her six-month evaluation, she discontinued the clinical trial due to brain fog and somnolence, which she reported significantly affected her ADLs beyond her baseline migraine symptoms. She stated her symptoms were resolved immediately after withdrawing from the clinical trial. Furthermore, her main symptom of nausea persisted, and she noted that the only relief she has received from nausea has been from OMT. At her six-month evaluation, the patient reported receiving additional OMT visits at irregular time intervals. She reported, “*...feeling the OMT working almost immediately, and the duration of effect varies depending on the severity of the migraine. I’ve had some sessions that take it away completely. Other sessions have reduced symptom severity, and the migraine comes back the next day.*” Finally, she reported that compared to her original medication management of propranolol, duloxetine Hcl (Cymbalta), and sumatriptan (Imitrex), the only intervention that had a complete abortive effect on migraine episodes and on her principal symptom of nausea was OMT. 

## Discussion

The integration of OMT into migraine management protocols has the potential to alleviate the physical and financial burden of this medical condition by offering patients a way to enhance their quality of life while evolving the landscape of migraine research and treatment. It is important to emphasize the cost burden of migraine management. The estimated unadjusted total healthcare expenditures for people with migraines is $56.31 billion, and the adjusted incremental total direct healthcare expenditure for those with migraines versus those without migraines is $9.21 billion per year [7,8]. One potential factor to consider regarding the high cost of medical management for migraines is the need for changing preventive pharmaceuticals due to variable efficacy. This case presents how one patient's previous standard of care was ineffective at managing her symptoms, and enrollment in a clinical trial for a novel pharmaceutical intervention also failed to produce adequate amelioration of migraine symptoms. This scenario reinforces the finding that 77.1% of patients make at least one change to their preventive migraine medication list or discontinue treatment [7]. Multiple reasons may be inferred for the necessity of medication change or discontinuation, such as adverse effects, lack of efficacy, or inability to treat the root cause of symptoms. It has been established that migraine causes are multifactorial, including genetic hereditary patterns, inflammatory mediators, and neurological mechanisms [9]. Musculoskeletal imbalances may also mediate migraines, as evidenced by this patient’s success with OMT. Additionally, one pilot randomized control trial identified a statistically significant decrease in analgesic consumption by targeting myofascial dysfunctions in chronic migraine patients [10]. Due to the multifaceted nature of chronic migraines, the integration of OMT alongside current pharmaceutical options creates a comprehensive treatment plan. 

The limitations of this case include the patient’s complex medication history and participation in a clinical trial, which contributed to an increase in headache symptoms. Additionally, the findings cannot achieve statistical significance due to the unique nature of this single case. Future research should focus on evaluating OMT both as a standalone therapy and in combination with pharmacological approaches. This would help further establish OMT's efficacy and enhance treatment options for patients.

To improve the generalizability and precision of findings, future clinical studies should include larger sample sizes with sufficient statistical power and the addition of a control group. This approach would support broader applicability, increase the validity of results, and help identify specific patient subgroups that may receive the greatest benefit from OMT, enabling more targeted treatment strategies. Additionally, it is important to note that future studies should include objective data measures separate from subjective data given the potential for bias. Furthermore, crossover studies could provide valuable insights by allowing participants to undergo multiple treatment modalities, separated by washout periods. Such studies could clarify the relative effectiveness of different approaches and determine the optimal treatment schedule and frequency of OMT and pharmacological treatments for managing chronic migraines.

## Conclusions

This case report highlights the efficacy of OMT as an intervention for treating chronic migraines. The results showed that using OMT to release physiological restrictions resolved the patient’s migraine symptoms for approximately one month. The patient said this treatment had longer-lasting results than her previous standard of care. The incorporation of OMT techniques such as cranial therapy, counterstrain, and HVLA may not only assist with managing migraine frequency and the associated pain but also decrease the usage of medications. Therefore, OMT may be used as an adjunct therapy to pharmacological medication or, in this case, as a stand-alone treatment, given the patient's contraindication to medication before beginning a clinical trial. While conventional medications have been the primary approach for symptom management, OMT introduces a complementary aspect that considers the interconnectedness of the body’s systems. Further studies are needed to evaluate the direct effects of OMT on migraines and other types of headaches, as well as to explore different treatment combinations. 
